# Corin deficiency impairs cardiac function in mouse models of heart failure

**DOI:** 10.3389/fcvm.2023.1164524

**Published:** 2023-08-11

**Authors:** Yayan Niu, Tiantian Zhou, Shengnan Zhang, Wenguo Li, Kun Wang, Ningzheng Dong, Qingyu Wu

**Affiliations:** ^1^ Cyrus Tang Hematology Center, Collaborative Innovation Center of Hematology, State Key Laboratory of Radiation Medicine and Prevention, Medical School, Soochow University, Suzhou, China; ^2^ NHC Key Laboratory of Thrombosis and Hemostasis, Jiangsu Institute of Hematology, The First Affiliated Hospital of Soochow University, Suzhou, China

**Keywords:** cardiac function, cardiac fibrosis, corin, heart failure, mouse models

## Abstract

**Introduction:**

Corin is a protease in the natriuretic peptide system. Deleterious *CORIN* variants are associated with hypertension and heart disease. It remains unclear if and to what extent corin deficiency may contribute to heart failure (HF).

**Methods:**

*Corin* knockout (KO) mice were used as a model. Cardiac function was assessed by echocardiography and tissue analysis in *Corin* KO mice at different ages or subjected to transverse aortic constriction (TAC), which increased pressure overload. Heart and lung tissues were analyzed for cardiac hypertrophy and lung edema using wheat germ agglutinin, Sirius red, Masson's trichrome, and Prussian blue staining. Recombinant corin was tested for its effect on cardiac function in the TAC-operated *Corin* KO mice. Selected gene expression in the heart was examined by RT-PCR. ELISA was used to analyze factors in plasma.

**Results:**

*Corin* KO mice had progressive cardiac dysfunction with cardiac hypertrophy and fibrosis after 9 months of age, likely due to chronic hypertension. When *Corin* KO mice were subjected to TAC at 10–12 weeks of age, cardiac function decreased more rapidly than in similarly treated wild-type mice. When the TAC-operated *Corin* KO mice were treated with recombinant corin protein, cardiac dysfunction, hypertrophy, and fibrosis were ameliorated. The corin treatment also decreased the gene expression associated with cardiac hypertrophy and fibrosis, increased plasma cGMP levels, lowered plasma levels of N-terminal pro-atrial natriuretic peptide, angiotensin II, and aldosterone, and lessened lung edema in the *Corin* KO mice subjected to TAC.

**Conclusion:**

Corin deficiency impairs cardiac function and exacerbates HF development in mice. Corin protein may be used to reduce cardiac hypertrophy and fibrosis, suppress the renin-angiotensin-aldosterone system, and improve cardiac function in HF.

## Introduction

Heart failure (HF) is a major disease characterized by the progressive loss of cardiac function (1). Atrial and B-type natriuretic peptides (ANP and BNP) are key hormones in the cardiac endocrine system that preserves body fluid balance and cardiac function (2–4). ANP also acts locally in the heart to regulate cellular homeostasis, preventing cardiac hypertrophy (5–7). In failing hearts, upregulation of natriuretic peptide expression is a compensatory mechanism to counter body fluid retention and maintain cardiomyocyte morphology and function. In HF patients, elevated plasma levels of unprocessed natriuretic peptides are well documented (8, 9), suggesting that defects in natriuretic peptide processing may be an importance underlying mechanism in the pathogenesis of HF.

Corin is a transmembrane serine protease expressed in the heart and non-cardiac tissues, where it converts the ANP precursor, pro-ANP, to mature ANP (10–14). The corin function is essential for body fluid-electrolyte balance and tissue homeostasis. In mice, corin deficiency prevents ANP generation, causing sodium retention, salt-sensitive hypertension, and cardiac hypertrophy (15–17). In the pregnant uterus, corin-mediated ANP activation is a key mechanism in endometrium decidualization and spiral artery remodeling, which is essential for maternal and fetal health (18, 19). In cell-based experiments, corin also activated BNP (20–22). This function, however, is dispensable *in vivo* (23), where furin, a proprotein convertase, has been identified as a primary enzyme for pro-BNP processing in cardiomyocytes (20, 24, 25).

To date, variants in the *NPPA* gene, encoding prepro-ANP, have been shown to be a key determinant in blood pressure levels and risks for cardiovascular disease (26–29). Similarly, variants in the *CORIN* gene have been reported in individuals with hypertension and heart disease (30–34). In HF patients, *CORIN* variants with impaired activity are associated with poor clinical outcomes (35). Circulating corin levels have also been identified as biomarkers in stroke, coronary artery disease, myocardial infarction, and HF (36–46). These findings suggest that defects in corin expression and/or function may be an underlying mechanism in heart disease. It remains unclear if and to what extent corin defects may contribute to the development of HF.

In this study, we examined the role of corin deficiency in the pathogenesis of HF. We used *Corin* knockout (KO) mice as an animal model to examine if corin deficiency exacerbates HF, particularly under pathological conditions. We studied cardiac morphology and function in *Corin* KO mice at different ages or subjected to transverse aortic constriction (TAC) that induces HF. We also tested if recombinant corin could be used as a therapeutic agent to improve cardiac function in *Corin* KO mice. Our findings should help to define the role of corin deficiency in the pathophysiology of heart disease.

## Materials and methods

### Animal ethical approval

All experimental procedures conducted in this study were approved by the Animal Use and Ethics Committee of Soochow University (201603A181).

### Corin KO mice

The strain of the *Corin* KO mice was published (17, 47). Briefly, two loxP loci were inserted in the *Corin* gene, flanking exon 4, to generate *Cor^flox^* mice, which were crossed with *CMV-Cre* mice expressing *Cre* in all tissues. The resultant mice with the null *Corin* allele were verified by DNA genotyping and analysis of *Corin* mRNA and protein expression by RT-PCR and western blotting, respectively (17, 47). The *Corin* KO mice were hypertensive [systolic blood pressure: ∼118 vs. ∼107 mm Hg in wild-type (WT) mice] and bred into the C57BL/6J background, as reported previously (17, 47). High levels of blood pressure in the *Corin* KO mice remained similar at different ages (Supplementary Figure S1). The *Corin* KO and C57BL/6J WT mice were housed at a temperature controlled specific-pathogen-free facility with 12:12-h light-dark cycles and ventilated cages containing bedding materials. The mice had free access to water and a regular chow diet with 0.59% NaCl.

### TAC-induced HF in mice

A TAC model was performed to induce HF in mice, as described previously (48). Briefly, *Corin* KO and C57BL/6 WT mice (male; 10–12 weeks old) (*n* = 13–15 per group) were anesthetized with isoflurane in oxygen (1.5%; flow rate of 0.3 L/min). The aortic arch and a 27-gauge needle were tied with a 7-0 silk suture at the level between the brachiocephalic trunk and the left carotid artery. As a sham control, similar surgical procedures were carried out except the suture constriction. Buprenorphine (0.1 mg/kg) was given to the mice as post-surgery analgesia up to 48 h. Echocardiography was conducted weekly to evaluate cardiac function, as described below. The experiment lasted 8 weeks.

### Analysis of cardiac function and morphology

Echocardiograph was carried out to evaluate cardiac function (49). Briefly, mice were anesthetized via inhalation of 1.5% isoflurane in oxygen (flow rate: 0.3 L/min). Transthoracic echocardiogram was conducted using VisualSonics equipment (Vevo2100) with a 30-MHz probe. Representative echocardiographic images were taken and shown in Supplementary Figure S2. Each echocardiographic measurement was done in triplicate. The data were analyzed by computer software (Vevo2100, VisualSonics). To assess cardiac hypertrophy, isolated hearts were weighed, and the data were normalized to body weights and tibia lengths.

### Histological examination

Mice were euthanized by exsanguination after isoflurane inhalation. Blood samples were collected in anti-coagulated tubes and centrifuged at 2,095*g* for 10 min at room temperature to obtain plasma. Tissues, including hearts and lungs, were dissected, weighed, and used for gene expression studies. For histological analysis, the tissues were treated with 4% (v/v) paraformaldehyde and embedded in paraffin. Tissue sections at 4 μm in thickness were prepared and stained with hematoxylin and eosin (H&E) (for general histology), rhodamine-conjugated wheat germ agglutinin (WGA) (Vector Laboratories, RL-1022-5) (for cardiac sarcolemma) or Sirius red and Masson's trichrome (Solarbio, G1340) (for fibrosis). To examine cardiomyocyte sizes, five randomly selected fields in each WGA-stained section were analyzed in a blinded manner. At least three sections were examined for each mouse. Diameters of at least 200 cardiomyocytes at short axis were measured at the nucleus plane with Image-Pro-Plus software (48). To evaluate cardiac fibrosis, percentages of Masson's trichrome-positive area in left ventricular (LV) sections were analyzed using Image-Pro-Plus software (Media Cybernetics, V6.0). To analyze lung edema, isolated lungs were weighed, and the data were normalized to body weights and tibia lengths. Lung sections were stained with Prussian blue (BBI Life Sciences, e670108) to assess iron-containing macrophages.

### Recombinant corin treatment

To test the effect of exogenous corin on cardiac function in *Corin* KO mice, a soluble form of corin (sCorin) was prepared, consisting of the entire extracellular region of human corin (residues 124-1042) and a C-terminal V5 tag (48). sCorin also contained an engineered zymogen activation site that can be cleaved by enterokinase (EK) (48). sCorin was expressed in Chinese hamster ovary (CHO)-K1 cells (NingBoMingZhou Tech, China) and purified from the conditioned medium with an affinity column, as described previously (48). The purified sCorin was quantified using a Bradford assay kit (Thermo Fisher Scientific, 23236) and activated by recombinant EK (BBI Life Sciences, C620005) (48). *Corin* KO mice were subjected to TAC or sham procedures. After 1 week, the mice with the TAC procedure were injected with sCorin (3 mg/kg, *i.p.*, daily) or equal volume of vehicle (Supplementary Figure S3). Cardiac function was assessed weekly by echocardiography. As reported previously (48), the intraperitoneal injection resulted in >20 µg/ml of sCorin in plasma with a half-life >8 h. After 7 weeks, the mice were euthanized. Blood and tissue samples were used for further gene and protein analyses.

### Cardiac gene expression

At 8 weeks post-TAC or sham operation, LV tissues were isolated from the mice without or with sCorin or vehicle treatment. Total RNAs were isolated from tissue homogenates with Trizol reagents (Ambion, 15596018) and used to make cDNAs using reverse transcriptase (Thermo Fisher Scientific, K1622). Quantitative RT-PCR (Thermo Fisher Scientific, 4485689) was used to examine expression levels of selected genes, including *Nppa* (encoding the ANP precursor), *Nppb* (encoding the BNP precursor), *Myh7* (encoding myosin heavy chain-β), and *Ctgf* (encoding connective tissue growth factor). The sequences of the oligonucleotide primers used in the RT-PCR are listed in Supplementary Table S1.

### Measurements of plasma factors

Plasma samples were prepared from the mice 8 weeks after sham or TAC procedures. Plasma levels of cGMP (Enzo Life Sciences, ADI-900-013; Shanghai MLBIO Biotechnology, ml001887), ANP (Shanghai Animalunion Biotechnology, LV30662), N-terminal (NT)-pro-ANP (Cloud-Clone Corp, SEA484Mu), angiotensin II (RayBio, EIAM-ANGII-1), and aldosterone (Elabscience, E-EL-0070c) were examined by ELISA. In the NT-pro-ANP ELISA, the antibody was raised against a pro-ANP fragment (Asn25-Arg122). Experimental procedures for the ELISA measurements were based on manufacturers' instructions.

### Statistics

Statistical analysis was done using Graphpad Prism 8.0 software (San Diego, CA, USA). Data were tested for normality and equal variance with Anderson-Darling, D'Agostino–Pearson, Kolmogorov–Smirnov, and Shapiro–Wilk tests. If passed, comparisons were done with Student's *t* test between two groups or one-way ANOVA followed by Tukey's *post hoc* analysis among three or more groups. Otherwise, the Kruskal–Wallis test was used. Quantitative data are presented in mean ± SEM. *P* values <0.05 were considered as statistically significant.

## Results

### Cardiac function in *Corin* KO mice at different ages

**Figure 1:**
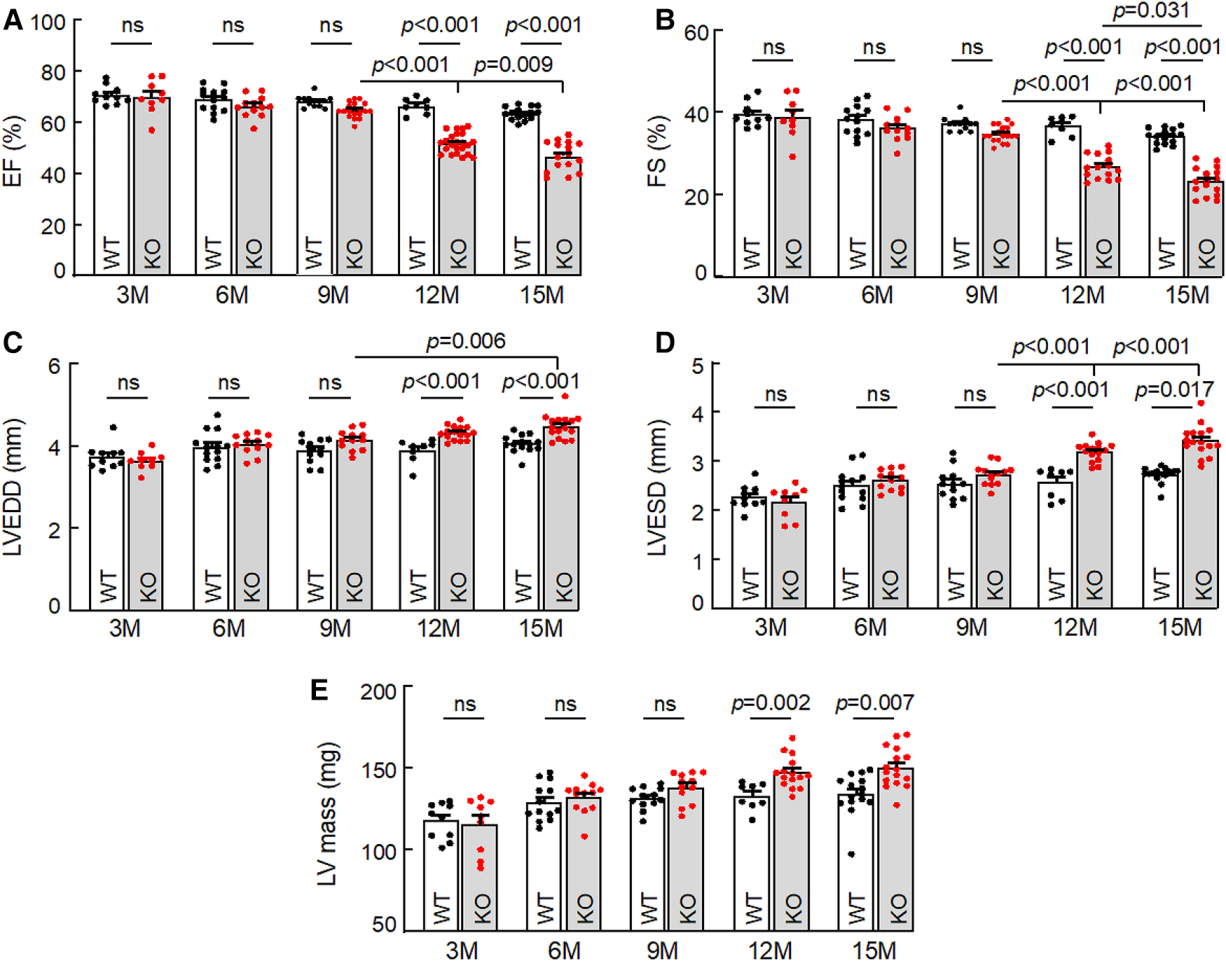
Cardiac function in WT and *Corin* KO mice at different ages. Echocardiography was conducted to examine ejection fraction (EF) (**A**), fractional shortening (FS) (**B**), left ventricular end diastolic dimension (LVEDD) (**C**), LV end systolic dimension (LVESD) (**D**), and LV mass (**E**) in male WT and *Corin* KO mice between 3 and 15 months of age. Data are mean ± SEM; *n* = 8–23 per group. *P* values were analyzed by one-way ANOVA and Tukey's *post hoc* analysis (**A–C,E**) or Kruslal-Wallis test (**D**). ns, not significant.

To examine the effect of corin deficiency on cardiac morphology and function, we performed echocardiography in WT and *Corin* KO mice at different ages. When tested between 3 and 9 months of age, male WT and *Corin* KO mice had similar values in parameters of cardiac function, including ejection fraction (EF) (Figure 1A), fractional shortening (FS) (Figure 1B), LV end diastolic dimension (LVEDD) (Figure 1C), LV end systolic dimension (LVESD) (Figure 1D), and LV mass (Figure 1E). When tested at 12 and 15 months of age, male WT mice had no apparent decline in cardiac function, whereas male *Corin* KO mice had reduced EF and FS and increased LVEDD, LVESD, and LV mass, compared to those in *Corin* KO mice at younger ages or in WT mice at similar ages (Figures 1A–E). Similar findings were also observed in female WT and *Corin* KO mice (Supplementary Figure S4), indicating that the effect of corin deficiency on cardiac function is gender independent.

**Figure 2:**
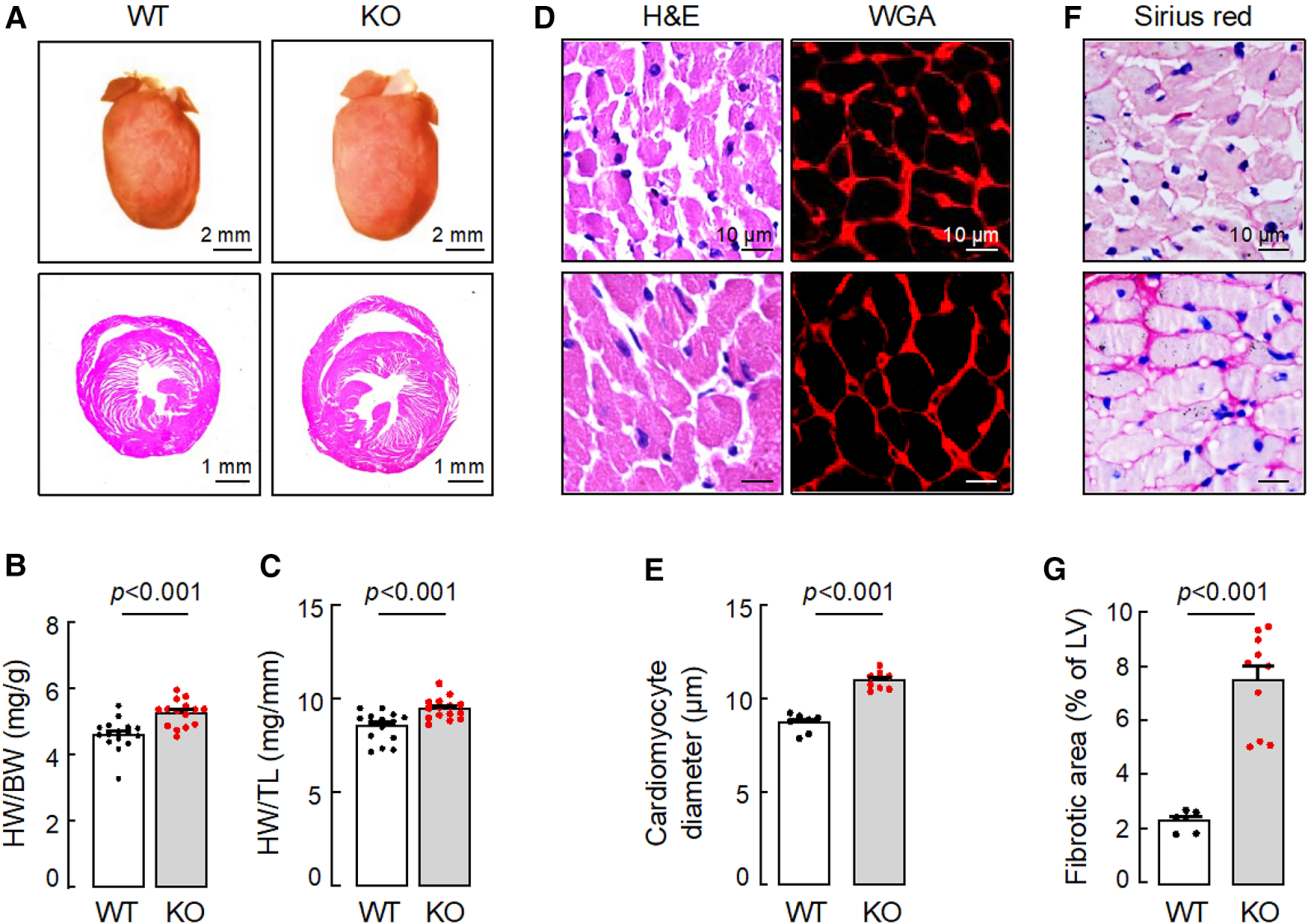
Cardiac hypertrophy and fibrosis in *Corin* KO mice. Hearts were isolated from 15-month-old male WT and *Corin* KO mice. Tissue sections were stained with hematoxylin and eosin (H&E), wheat germ agglutinin (WGA), or Sirius red. (**A**) Representative pictures of hearts and H&E-stained sections from WT and *Corin* KO mice are shown. (**B,C**) Heart weights (HW) were normalized to body weights (BW) (**B**) or tibia length (TL) (**C**). (**D,E**) Cardiomyocyte diameters in H&E- or WGA-stained LV sections were measured at the nucleus plane in 200 individual cells randomly selected from three sections. At least three sections per mouse were analyzed. (**F,G**) Connective tissues (purple) in at least five randomly selected fields from Sirius red-stained LV sections were analyzed. Percentages of fibrotic area were calculated using Image-Pro-Plus software. Scale bars are indicated. Quantitative data are mean ± SEM analyzed by Student's *t* test.

In tissue analysis, hearts from 15-month-old *Corin* KO mice appeared larger (Figure 2A) and were heavier than those from age-matched WT mice, as indicated by greater heart weights (HW), normalized to body weights (BW) (Figure 2B) or tibia lengths (TL) (Figure 2C) (Supplementary Table S2). In H&E- and WGA-stained heart sections, cardiomyocytes from the *Corin* KO mice were larger in diameters, compared to those from WT mice (Figures 2D,E). Sirius red staining revealed increased fibrosis in heart sections from the *Corin* KO mice, whereas no apparent fibrosis was detected in heart sections from the aged-matched WT mice (Figures 2F,G). These results indicate that *Corin* KO mice at 12 months of age or older developed cardiac hypertrophy, cardiac fibrosis, and HF.

### Exacerbated HF in *Corin* KO mice subjected to TAC

**Figure 3:**
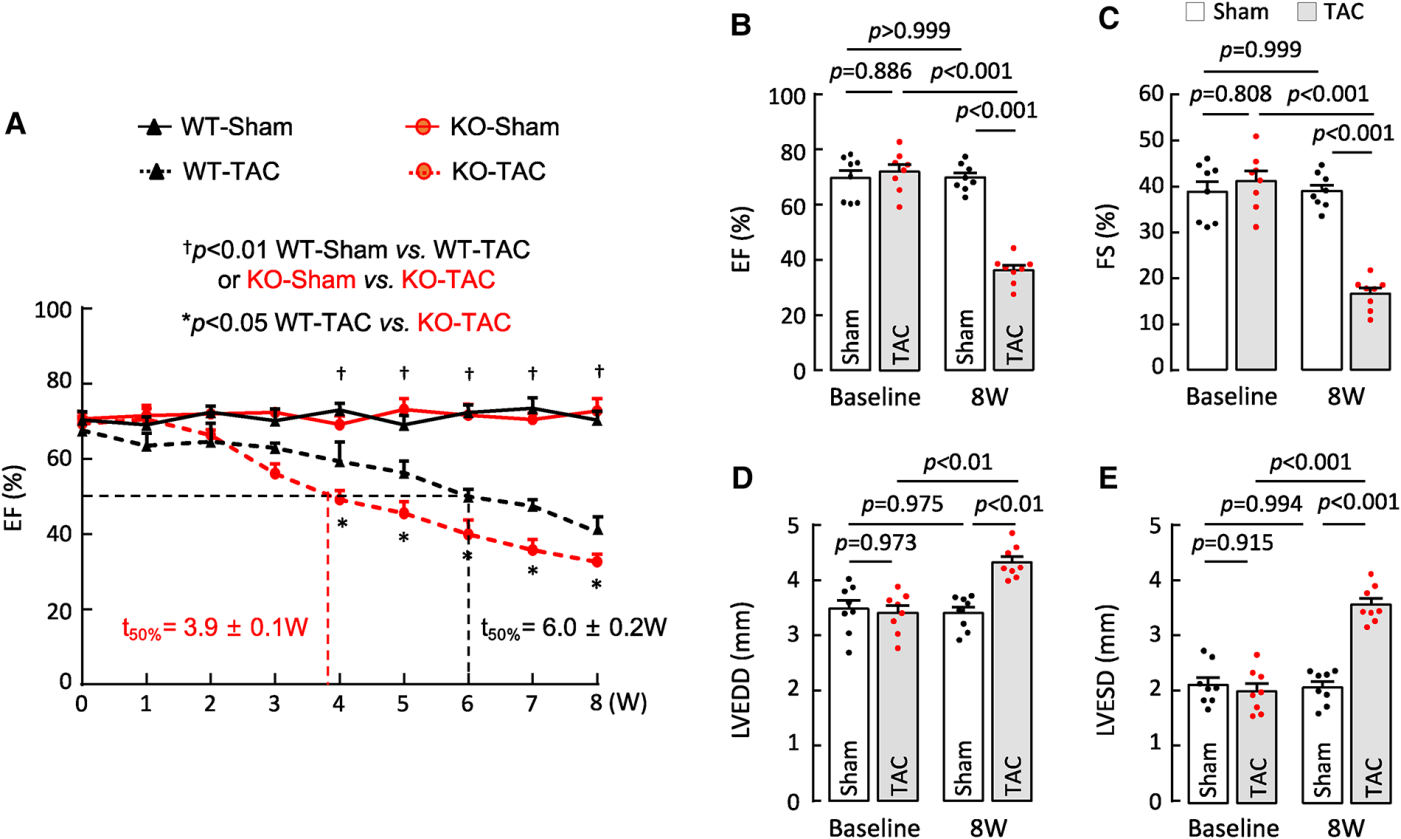
Cardiac function in *Corin* KO mice subjected to TAC. TAC was done in 10–12-week-old male WT and *Corin* KO mice (*n* = 8 per group). Cardiac function was assessed weekly with echocardiography. (**A**) EF in sham- or TAC-operated WT and *Corin* KO mice after the surgery. Comparisons were done by two-way ANOVA and Tukey's *post hoc* analysis between sham- or TAC-operated WT or *Corin* KO mice and between TAC-operated WT and *Corin* KO mice. Data are mean ± SEM; *n* = 8 per group. Times when EF values were 50% (*t*_50%_) in WT (black) and *Corin* KO (red) mice were calculated. (**B–E**) EF (**B**), FS (**C**), LVEDD (**D**), and LVESD (**E**) in sham- or TAC-operated *Corin* KO mice before (baseline) and 8 weeks post-surgery were examined by one-way ANOVA and Tukey's *post hoc* analysis. Data are mean ± SEM; *n* = 8 per group.

**Figure 4:**
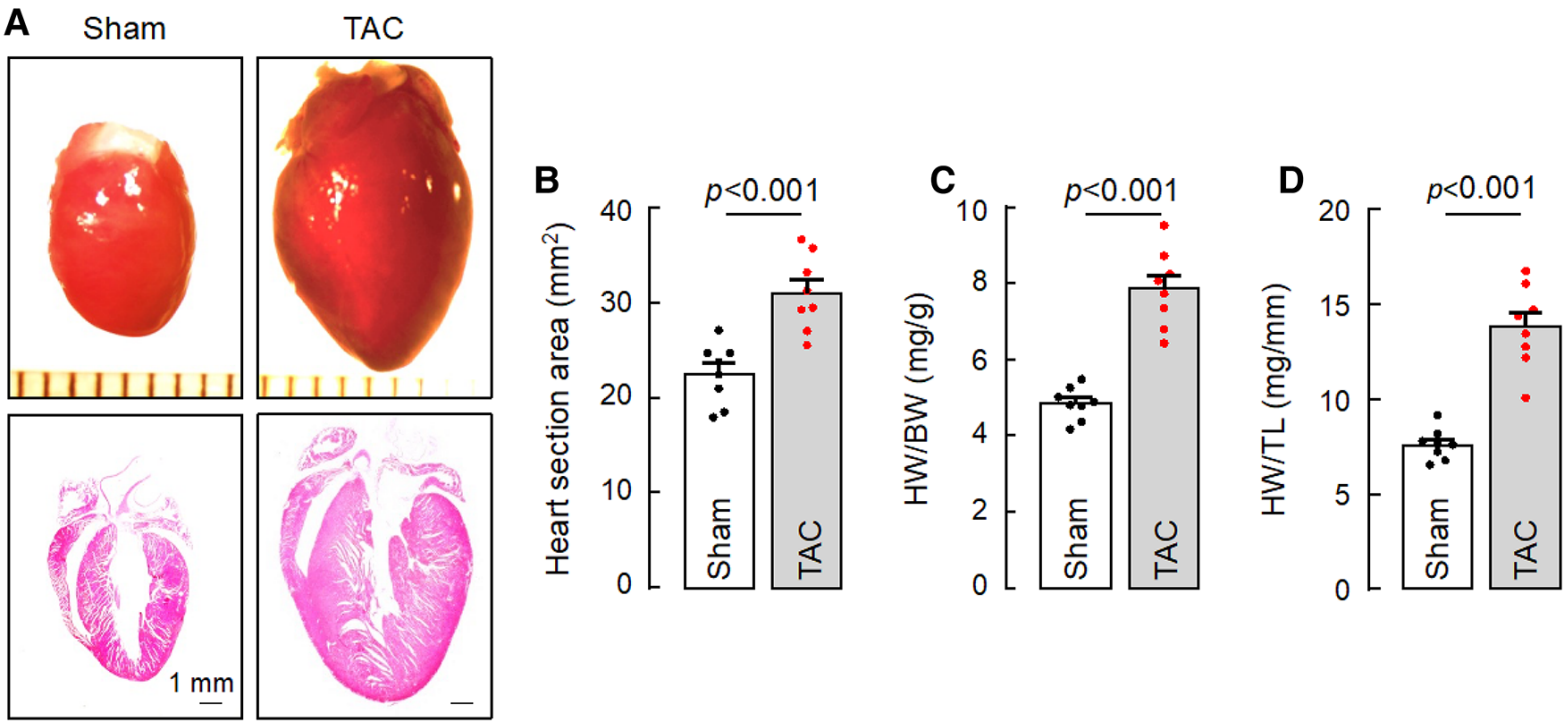
Cardiac hypertrophy in TAC-operated *Corin* KO mice. Hearts were isolated from sham- or TAC-operated *Corin* KO mice at 8 weeks post-surgery. Tissue sections were stained with H&E. (**A**) Representative pictures of hearts and H&E-stained sections from the sham- and TAC-operated *Corin* KO mice are shown. (**B**) Heart section areas were analyzed by Image-Pro-Plus software. (**C,D**) Values of HW were normalized to BW (**C**) or TL (**D**). Data in **B–D** are mean ± SEM analyzed by Student's *t* test.

HF may result from many pathological insults. To examine if corin deficiency exacerbates HF under pathological conditions, we tested a HF model induced by TAC in 10–12-week-old WT and *Corin* KO mice (Supplementary Figure S2). By echocardiography, we found that cardiac function decreased progressively after WT and *Corin* KO mice were subjected to TAC, whereas cardiac function remained unchanged in the corresponding mice subjected to sham operation (Figure 3A) (Supplementary Figure S5). The rate of the decline was much faster in the *Corin* KO than WT mice after TAC; the times for EF to fall below 50% was 3.9 ± 0.1 and 6.0 ± 0.2 weeks (*n* = 8, *P *< 0.001) in the *Corin* KO and WT mice, respectively (Figure 3A). At 8 weeks post-TAC, the *Corin* KO mice had markedly reduced EF (Figure 3B) and FS (Figure 3C) and increased LVEDD (Figure 3D) and LVESD (Figure 3E), compared to those in the *Corin* KO mice at the baseline or 8 weeks post-sham operation. In tissue and histological examinations, hearts isolated from the *Corin* KO mice at 8 weeks post-TAC were larger (Figures 4A,B) and heavier (Figures 4C,D) (Supplementary Table S3), compared with those from the corresponding sham-operated *Corin* KO mice or the TAC-operated WT mice (Supplementary Figure S6). These results indicate that corin deficiency accelerated cardiac hypertrophy and HF in mice upon aortic constriction.

### Improved cardiac function and morphology in *Corin* KO mice treated with sCorin

**Figure 5:**
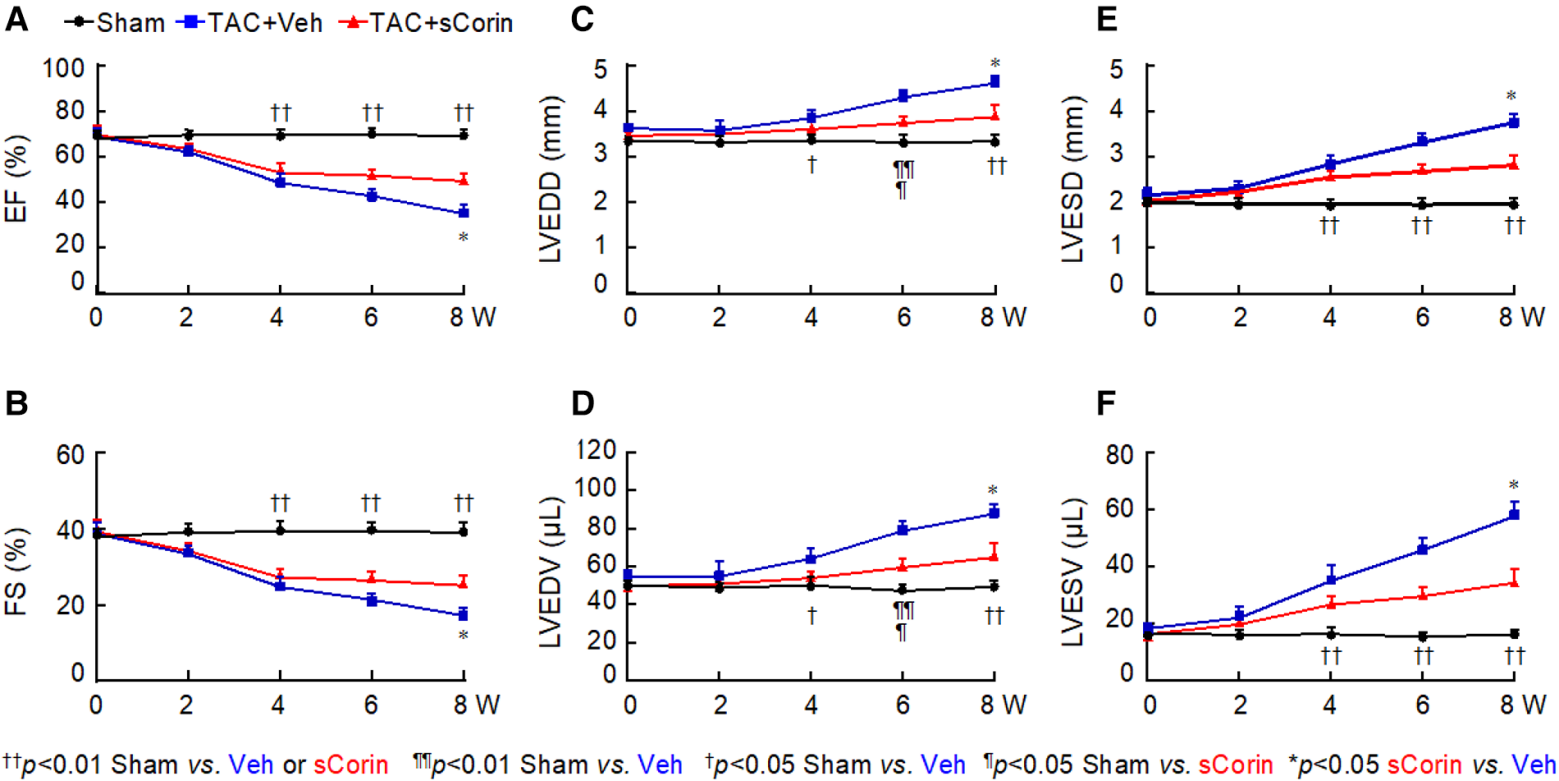
Cardiac function in TAC-operated *Corin* KO mice treated with sCorin. *Corin* KO mice (male, 10–12 weeks old) were subjected to sham or TAC operation (*n* = 13–15 per group). The TAC-operated mice were treated with a control vehicle (TAC + Veh) or sCorin (TAC + sCorin), starting at 1-week post-surgery. Cardiac function was assessed weekly with echocardiography. Data of EF (**A**), FS (**B**), LVEDD (**C**), LV end diastolic volume (LVEDV) (**D**), LVESD (**E**), and LV end systolic volume (LVESV) (**F**) were analyzed by one-way ANOVA and Tukey's *post hoc* analysis.

Previously, transgenic *Corin* expression in the heart or recombinant corin protein administration improved cardiac function in mouse models of HF (48, 50). We tested if a similar approach could improve cardiac function in *Corin* KO mice with TAC. We performed TAC or sham procedures in 10–12-week-old *Corin* KO mice, which had normal cardiac function, followed by weekly peritoneal injection of vehicle or a soluble form of corin (sCorin) consisting of the full length extracellular region of corin (48) (Supplementary Figure S3). The sCorin injection increased plasma ANP levels (Supplementary Figure S7). As indicated by echocardiographic measurements, including EF (Figure 5A), FS (Figure 5B), LVEDD (Figure 5C), LV end diastolic volume (LVEDV) (Figure 5D), LVESD (Figure 5E), and LV end systolic volume (LVESV) (Figure 5F), cardiac function declined progressively in the *Corin* KO mice subjected to TAC, compared to those in the sham-operated controls. After 7 weeks of sCorin injection, cardiac function was improved in the TAC-operated *Corin* KO mice, compared to that in the vehicle-treated corresponding controls (Figures 5A–F).

**Figure 6:**
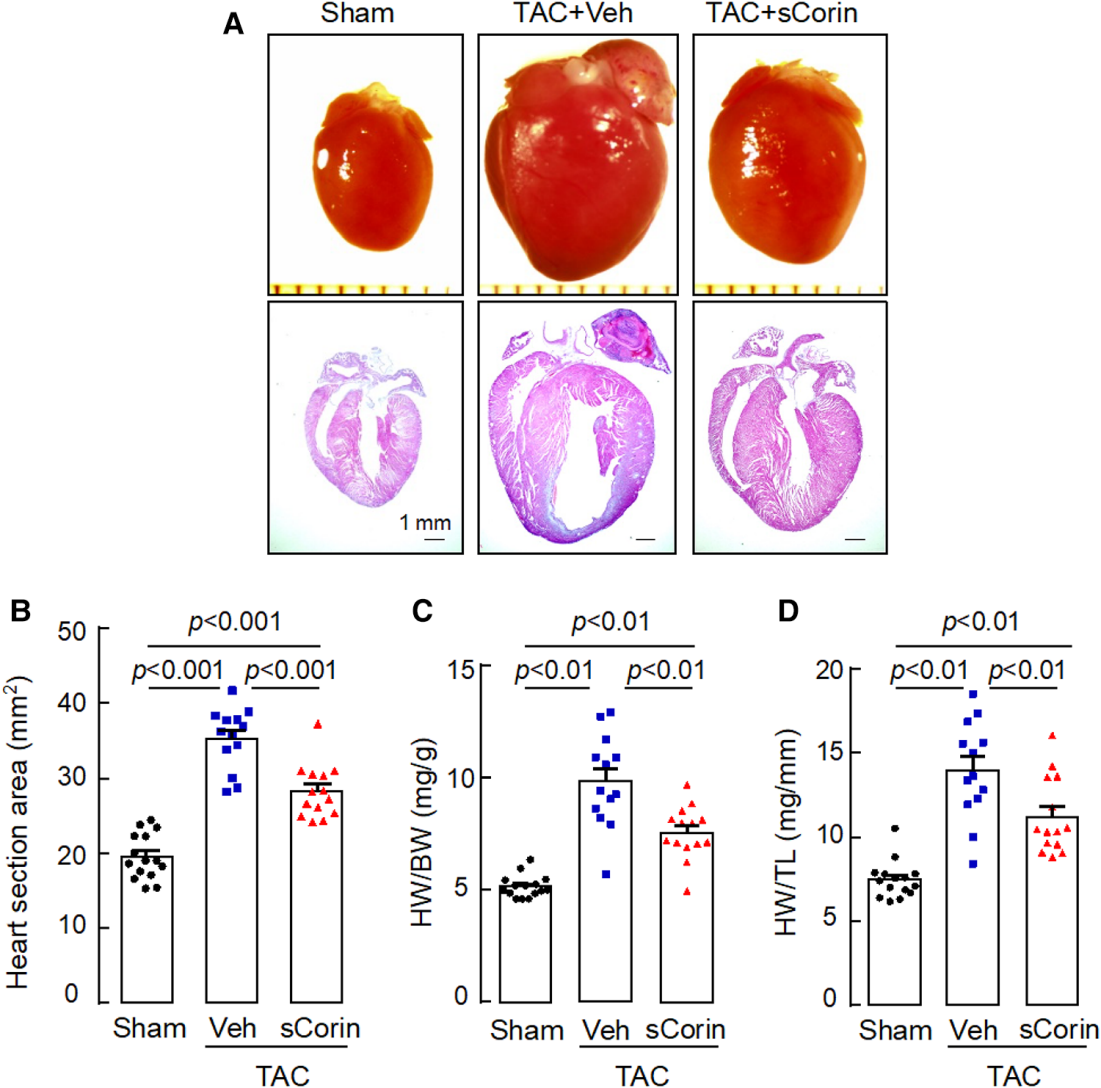
Increased heart size and weight in TAC-operated *Corin* KO mice treated with sCorin. Hearts were isolated from the sham- or TAC-operated *Corin* KO mice with vehicle (TAC + Veh) or sCorin (TAC + sCorin) treatment at 8 weeks post-surgery. Tissue sections were stained with H&E. (**A**) Representative pictures of hearts and H&E-stained sections from the sham- and TAC-operated *Corin* KO mice are shown. (**B**) Heart section areas were analyzed by Image-Pro-Plus software. (**C,D**) Values of HW were normalized to BW (**C**) or TL (**D**). Data in **B–D** are mean ± SEM analyzed by one-way ANOVA and Tukey's *post hoc* analysis.

Analysis of hearts isolated at 8 weeks post-TAC showed that cardiac hypertrophy occurred in the TAC-operated *Corin* KO mice, compared to the sham-operated mice (Figures 6A–D). In the TAC-operated *Corin* KO mice that received sCorin injection, cardiac hypertrophy was markedly reduced, as indicated by smaller hearts (Figures 6A,B) and reduced ratios of HW to BW (Figure 6C) or TL (Figure 6D) (Supplementary Table S4), compared to those in the TAC-operated *Corin* KO mice receiving the vehicle.

**Figure 7:**
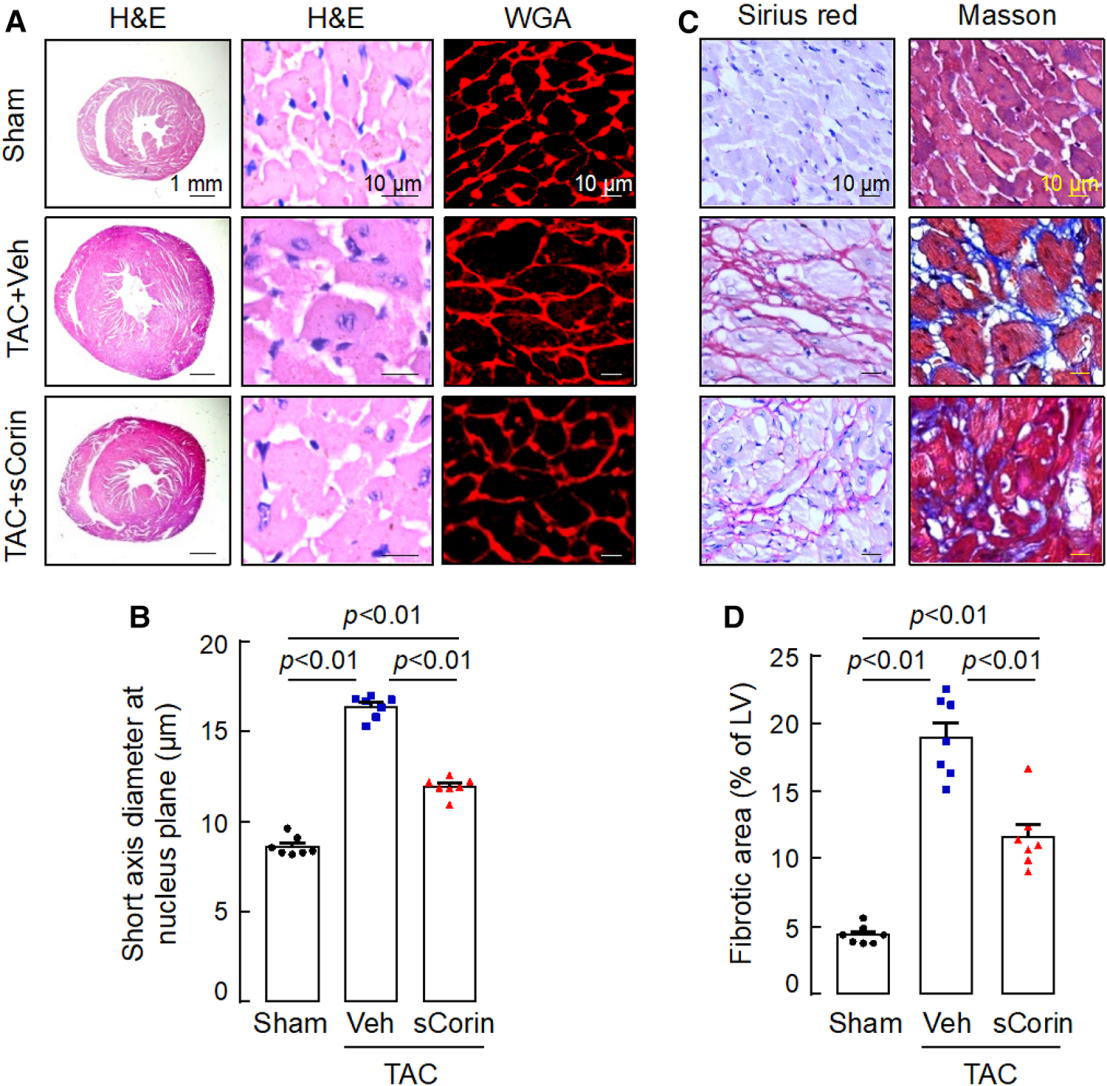
Cardiac hypertrophy and fibrosis in TAC-operated *Corin* KO mice treated with sCorin. *Corin* KO mice (male, 10–12 weeks old) were subjected to sham or TAC operation. The TAC-operated *Corin* KO mice were treated with a control vehicle (TAC + Veh) or sCorin (TAC + sCorin). Hearts were isolated at 8 weeks post-surgery (*n* = 7 per group). (**A,B**) To assess cardiac hypertrophy, heart sections were stained with H&E and WGA (**A**). Diameters of at least 200 cardiomyocytes in randomly selected LV sections were measured at the nucleus plane (**B**). At least three sections per mouse were analyzed. (**C,D**) To assess cardiac fibrosis, heart sections were stained with Sirius red and Masson's trichrome (**C**). Fibrotic areas in at least three randomly selected LV section fields were analyzed by Image-Pro-Plus software (**D**). The data in (**B,D**) were analyzed by one-way ANOVA and Tukey's *post hoc* analysis.

Analysis of H&E- and WGA-stained heart sections revealed that cardiomyocytes in the TAC-operated *Corin* KO mice were larger in diameters, compared to those in the sham-operated *Corin* KO mice (Figures 7A,B). In the TAC-operated *Corin* KO mice with sCorin treatment, cardiomyocyte diameters were reduced, compared to those in the TAC-operated *Corin* KO mice with vehicle treatment (Figures 7A,B). Similarly, analysis of Sirius red- or Masson's trichrome-stained heart sections revealed increased fibrosis in the TAC-, but not sham-, operated *Corin* KO mice (Figures 7C,D). In the TAC-operated *Corin* KO mice with sCorin injection, the area of fibrosis was markedly reduced, compared to that in the vehicle-treated mice (Figures 7C,D). These results indicate that administration of exogenous corin protein reduced cardiac hypertrophy and fibrosis and improved cardiac function in the *Corin* KO mice subjected to TAC.

### Cardiac gene expression in *Corin* KO mice treated with sCorin

**Figure 8:**
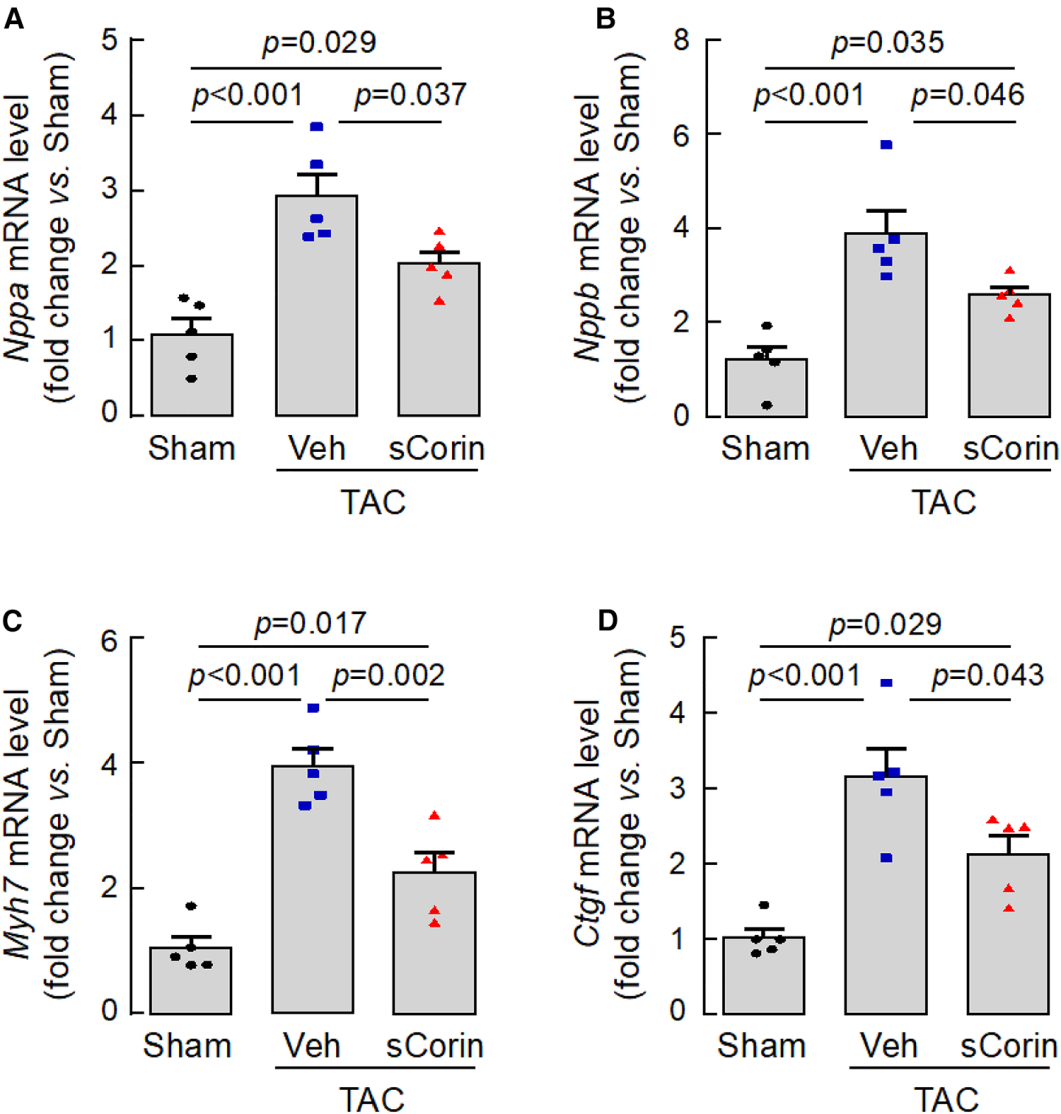
Cardiac gene expression in TAC-operated *Corin* KO mice treated with sCorin. Hearts were isolated from the sham- or TAC-operated *Corin* KO mice with vehicle (TAC + Veh) or sCorin (TAC + sCorin) treatment at 8 weeks post-surgery. Quantitative RT-PCR was done to assess expression levels of *Nppa* (**A**), *Nppb* (**B**), *Myh7* (**C**), and *Ctgf* (**D**), which are associated with cardiac hypertrophy and fibrosis (*n* = 5 per group). Data were analyzed by one-way ANOVA and Tukey's *post hoc* analysis.

To verify our findings, we analyzed a set of selected genes associated with cardiac hypertrophy and fibrosis in the *Corin* KO mice subjected to TAC. By quantitative RT-PCR, we found that hearts from the TAC-operated *Corin* KO mice had increased levels of *Nppa*, *Nppb*, *Myh7*, which were associated cardiac hypertrophy, and *Ctgf*, which was associated with cardiac fibrosis, compared with those in the sham-operated *Corin* KO mice (Figures 8A–D). The expression levels of these genes were reduced after the TAC-operated *Corin* KO mice received the sCorin treatment (Figures 8A–D). These results are consistent with the findings that the sCorin treatment reduced cardiac hypertrophy and fibrosis in the *Corin* KO mice subjected to TAC.

### Analysis of plasma factors in *Corin* KO mice treated with sCorin

**Figure 9:**
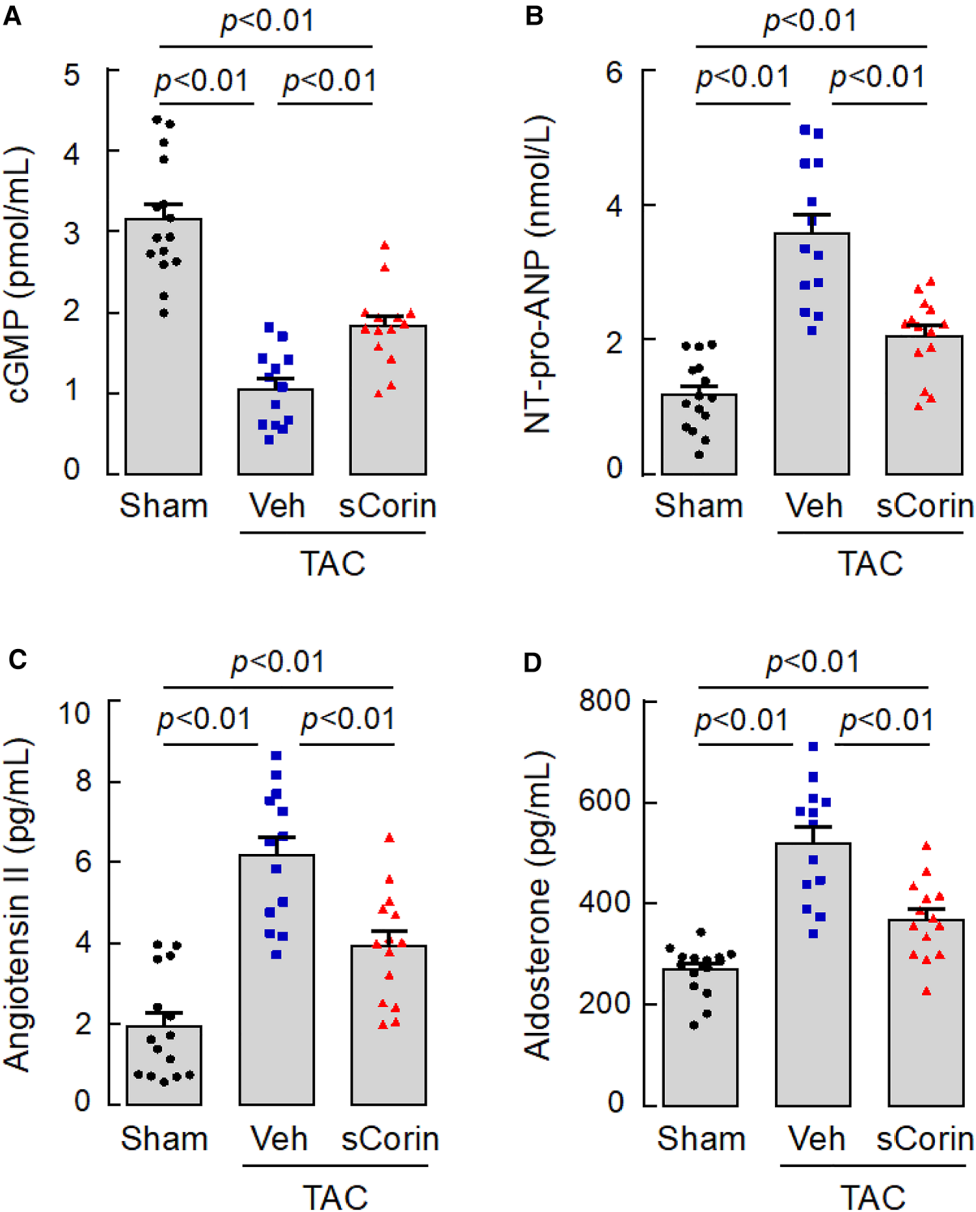
Changes of plasma factor levels in TAC-operated *Corin* KO mice treated with sCorin. Plasma samples were isolated from the sham- or TAC-operated *Corin* KO mice with vehicle (TAC + Veh) or sCorin (TAC + sCorin) treatment at 8 weeks post-surgery. Levels of cGMP (**A**), N-terminal (NT)-pro-ANP (**B**), angiotensin II (**C**), and aldosterone (**D**) were measured by ELISA (*n* = 13–15 per group). Data were analyzed by one-way ANOVA and Tukey's *post hoc* analysis.

We next measured plasma cGMP levels, an indicator of natriuretic peptide activity. We found reduced plasma cGMP levels in the TAC-operated *Corin* KO mice, compared to those in the sham-operated *Corin* KO mice (Figure 9A). The levels were increased after the TAC-operated *Corin* KO mice were treated with sCorin. In contrast, levels of plasma NT-pro-ANP, angiotensin II, and aldosterone were increased in the TAC-operated *Corin* KO mice, compared with those in the sham-operated mice. The levels were decreased after the TAC-operated *Corin* KO mice were treated with sCorin (Figures 9B–D). These results are consistent, indicating that the TAC-operated *Corin* KO mice had increased *Nppa* expression, impaired natriuretic peptide processing, and enhanced angiotensin and aldosterone levels and that such a phenotype was ameliorated after the sCorin treatment.

### Lessened lung edema in *Corin* KO mice treated with sCorin

**Figure 10:**
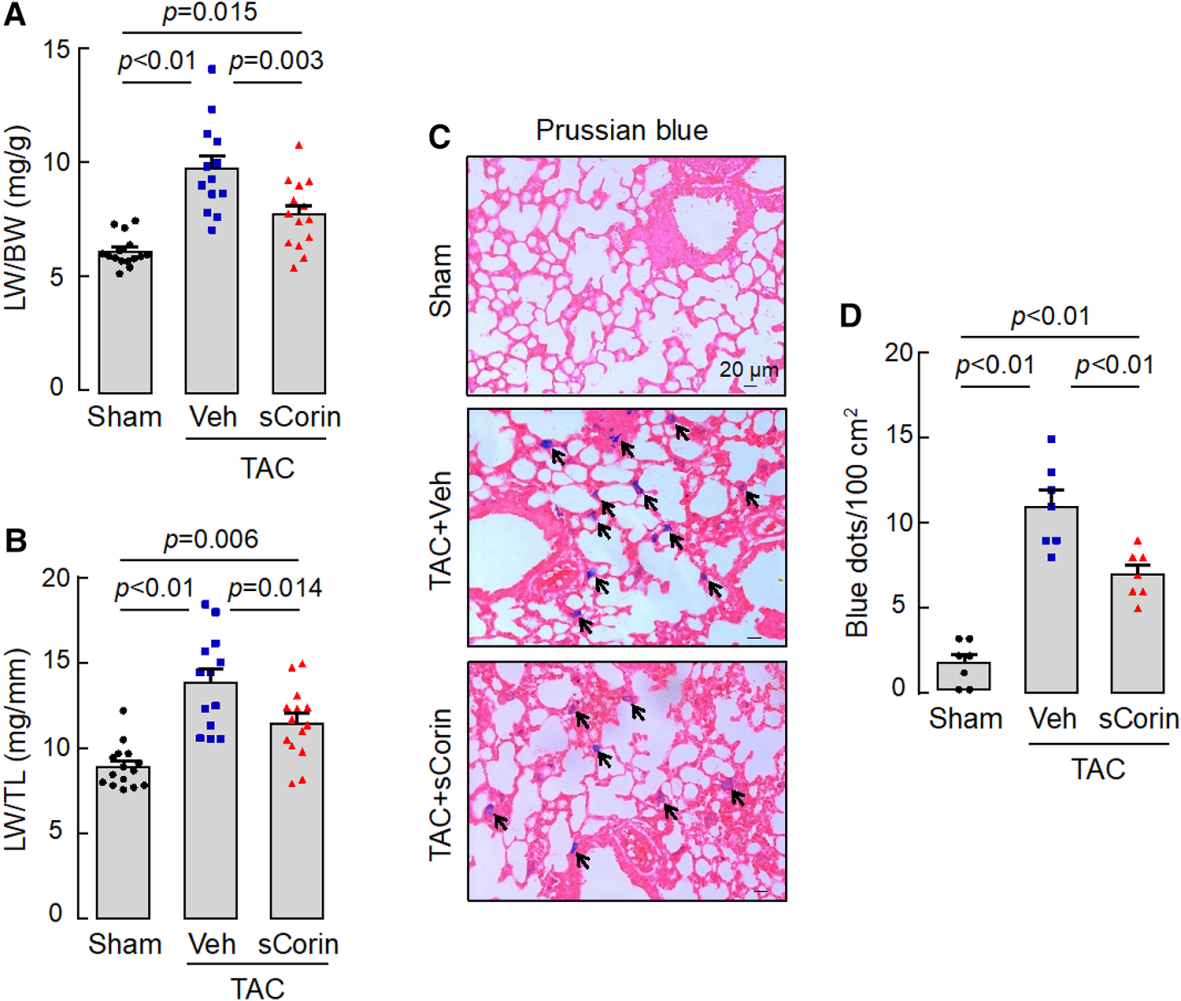
Analysis of lungs from TAC-operated *Corin* KO mice treated with sCorin. Lungs were isolated from the sham- or TAC-operated *Corin* KO mice with vehicle (TAC + Veh) or sCorin (TAC + sCorin) treatment at 8 weeks post-surgery. Lung weights (LW) were normalized to BW (**A**) or TL (**B**) (*n* = 13–15 per group). Lung sections were stained with Prussian blue for macrophages containing hemosiderin (blue dots) (**C**). Blue dots in at least three randomly selected fields from at least three sections per mouse were counted (*n* = 7 mice per group). Data in (**A,B,D**) were analyzed by one-way ANOVA and Tukey's *post hoc* analysis.

Lung congestion and edema are common pathological features in HF. Compared to the sham-operated mice, the TAC-operated *Corin* KO mice had increased lung weights (LW), normalized to BW or TL (Figures 10A,B). By Prussian blue staining for macrophages containing hemosiderin, we found more blue spots in lung sections from the TAC-operated *Corin* KO mice (Figures 10C,D). The lung weights and Prussian blue staining were reduced in the TAC-operated *Corin* KO mice after the sCorin treatment (Figures 10C,D). These results indicate that TAC-induced HF resulted in lung edema in the TAC-operated *Corin* KO mice, which was ameliorated after the sCorin treatment.

## Discussion

Hypertension is an important risk factor in the pathogenesis of HF (1, 51). Corin and ANP-mediated signaling is critical for preserving normal blood pressure. Deficiencies in *Corin* and *Nppa* genes cause salt-sensitive hypertension in mice (17, 52–54). In this study, we examined to what extent corin deficiency may impair cardiac function and morphology in mice with a null *Corin* allele. We found that the *Corin* KO mice had normal cardiac function up to 9 months of age, after which cardiac function declined progressively. The abnormal cardiac function was associated with cardiac hypertrophy and fibrosis, as indicated by echocardiography and histological analysis of heart tissues in the *Corin* KO mice of 12–15 months of age. In the *Corin* KO mice, blood pressure levels were continuously high, as measured between 3 and 15 months of age. These findings suggest that impaired cardiac function and morphology in the aged *Corin* KO mice may result primarily from chronic pressure overload, but not intrinsic structural defects in the heart. Previously, corin was reported to protect against ischemia and oxidative stress-induced apoptosis in cultured cardiomyocytes and neuronal cells (55, 56). Based on the findings in our study, it seems unlikely that enhanced cardiomyocyte apoptosis is a major cellular event in the *Corin* KO mice, at least up to 9 months of age without additional pathological challenges.

Consistent with the findings in the aged *Corin* KO mice, we found that TAC, which causes pressure overload (57, 58), led to a marked decline in cardiac function in 10–12-week-old *Corin* KO mice, indicating that corin deficiency exacerbates HF development upon aortic constriction. Previously, a strain of mast cell-deficient mice, i.e., C57BL/6-*Kit*^W−sh^ (W^sh^) mice, was reported to contain an inversion breakpoint in a genetic locus containing *Kit*, *Corin*, *Pdgfra* (encoding platelet-derived growth factor receptor-α), and other genes (59). The W^sh^ mice also exhibited a phenotype of cardiac hypertrophy (59) and, when subjected to TAC, a rapid decline in cardiac function (60), resembling our findings in *Corin* KO mice. Unlike in the *Corin* KO mice subjected to TAC, however, there was no detectable cardiac fibrosis in the W^sh^ mice after TAC (60). Mast cells are known to produce many proteases, cytokines, and growth factors that are important in immune responses, inflammation, and tissue homeostasis (61, 62). It remains to be determined if some of those mast cell-derived molecules are involved in tissue remodeling and cardiac fibrosis in failing hearts, which may account for the apparent difference in cardiac fibrosis between the mast cell-deficient W^sh^ mice and the *Corin* KO mice subjected to TAC.

Genetic variants that impair corin expression and/or function have been reported in patients with hypertension and cardiovascular disease (30–34). A *CORIN* variant allele (T555I/Q568P), for example, has been identified in ∼10%–12% African Americans (63, 64), a population with a high prevalence for hypertension and heart disease (65). Individuals with this *CORIN* allele had an increased cardiac hypertrophic response to pressure overload, as indicated by greater LV mass normalized to fat-free mass or body surface area as a function of systolic blood pressure (66, 67). In cell-based experiments, the corin variant T555I/Q568P was defective in proprotein convertase subtilisin/kexin-6 mediated zymogen activation and pro-ANP/pro-BNP processing (23, 68). In a transgenic model, mice expressing the corin variant also exhibited cardiac hypertrophy that was exacerbated by high-salt diets or during pregnancy (49, 52). These results are consistent with our findings in this study, suggesting that individuals with corin defects may be more susceptible to developing cardiac hypertrophy and HF, particularly in the presence of additional pathological challenges.

In patients with late stages of HF, body fluid retention is common, as manifested by shortness of breath, orthopnea, and low leg swelling (1). Approaches to enhance natriuretic peptide activity or to reduce natriuretic peptide degradation have been used to improve body fluid balance in individuals with failing hearts (69–72). In mouse models of myocardial infarction and cardiomyopathy, transgenic corin expression in the heart decreased cardiac dysfunction, lowered edema, and improved survival (50, 73, 74). Similarly, our recent studies also found that in WT mice, cardiac hypertrophy and dysfunction induced by left coronary artery ligation or TAC were ameliorated by administration of recombinant sCorin (48). In this study, we showed that sCorin injection improved cardiac function, reduced cardiac hypertrophy and fibrosis, and lessened lung edema in the *Corin* KO mice subjected to TAC. Moreover, the sCorin treatment also led to changes in cardiac gene expression and circulating factors, including the reduced levels of *Nppa*, *Nppb*, *Myh7* and *Cfgf* expression in the heart, increased plasma cGMP levels, and suppressed plasma levels of NT-pro-ANP, angiotensin II, and aldosterone in these mice. These findings are consistent, indicating the improved cardiac morphology and function, which are associated with enhanced natriuretic peptide processing and signaling and suppressed renin-angiotensin-aldosterone system in the *Corin* KO mice treated with sCorin. In patients with HF, impaired natriuretic peptide processing is common, as indicated by elevated levels of circulating pro-ANP and pro-BNP (8, 9). Together, these results suggest that strategies to enhance corin activity may be explored to improve cardiac function in HF patients, particularly those with deficiencies in corin expression and/or activity.

In conclusion, we showed that the *Corin* KO mice had cardiac dysfunction beyond 12 months of age, which was likely caused by chronic hypertension. When subjected to TAC at a young age, the *Corin* KO mice exhibited worsening cardiac hypertrophy, cardiac fibrosis, HF, and lung edema, indicating that corin deficiency exacerbated HF development in the presence of additional pathological challenges. In the *Corin* KO mice subjected to TAC, administration of recombinant corin protein enhanced natriuretic peptide activity, suppressed the renin-angiotensin-aldosterone system, improved cardiac morphology and function, and lessened lung edema. These results indicate that corin deficiency is an underlying factor in the pathogenesis of HF, suggesting that recombinant corin protein may be used to improve the function of failing hearts in patients with impaired natriuretic peptide processing.

## Data Availability

The original contributions presented in the study are included in the article/**Supplementary Material**, further inquiries can be directed to the corresponding authors.
