# Supplementary material for: Corin deficiency impairs cardiac function in mouse models of heart failure
Source: Front Cardiovasc Med. 2023 Aug 11;10:1164524. doi: 10.3389/fcvm.2023.1164524 (PMC10450958; doi:10.3389/fcvm.2023.1164524)
Supplement: Supplementary file 1 [file Datasheet1.pdf]

Supplementary Figures and Tables

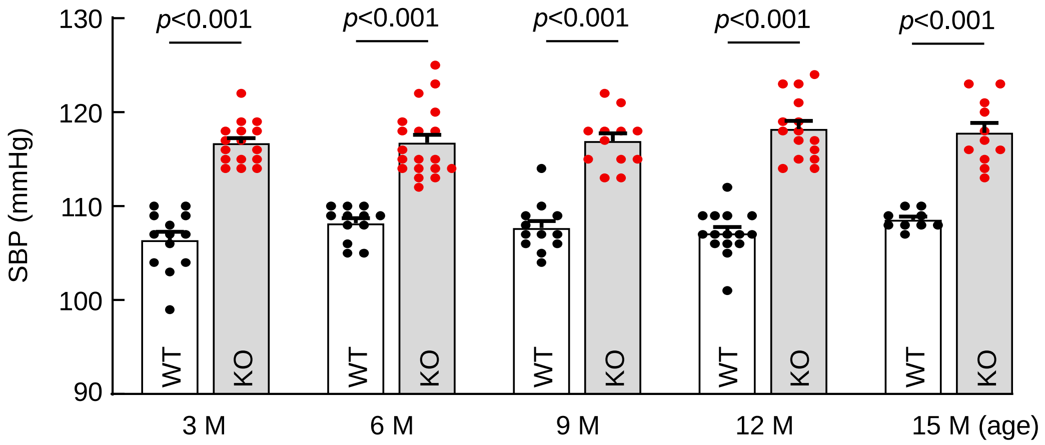

Supplementary Figure 1. Systolic blood pressure (SBP) in male WT and *Corin* KO mice at different months (M) of ages. Data are mean ± SEM. n = 9-19 per group. *P* values were analyzed by one-way ANOVA.

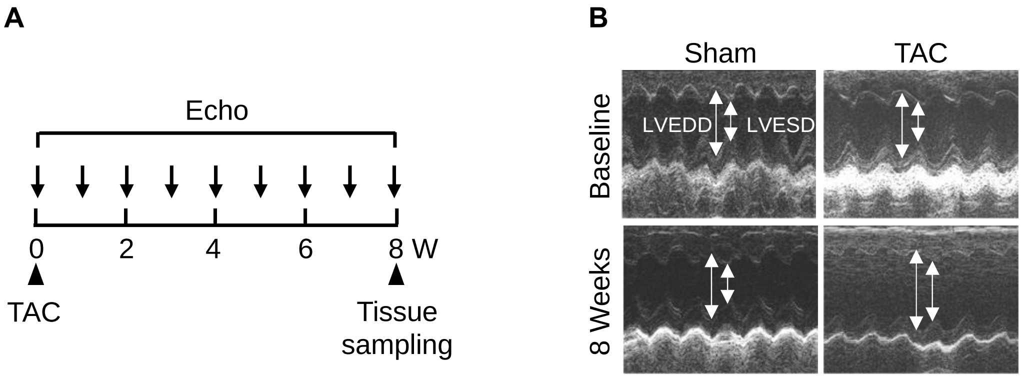

Supplementary Figure 2. Illustration of the TAC-induced heart failure model in mice. Sham or TAC operation was done in WT and *Corin* KO mice (male, 10-12 weeks old) (n = 8 per group). Echocardiography (Echo) was done weekly to assess cardiac function before (0 w) and up to eight weeks post-surgery, as illustrated in (**A**). Representative echocardiographic images are shown in (**B**).

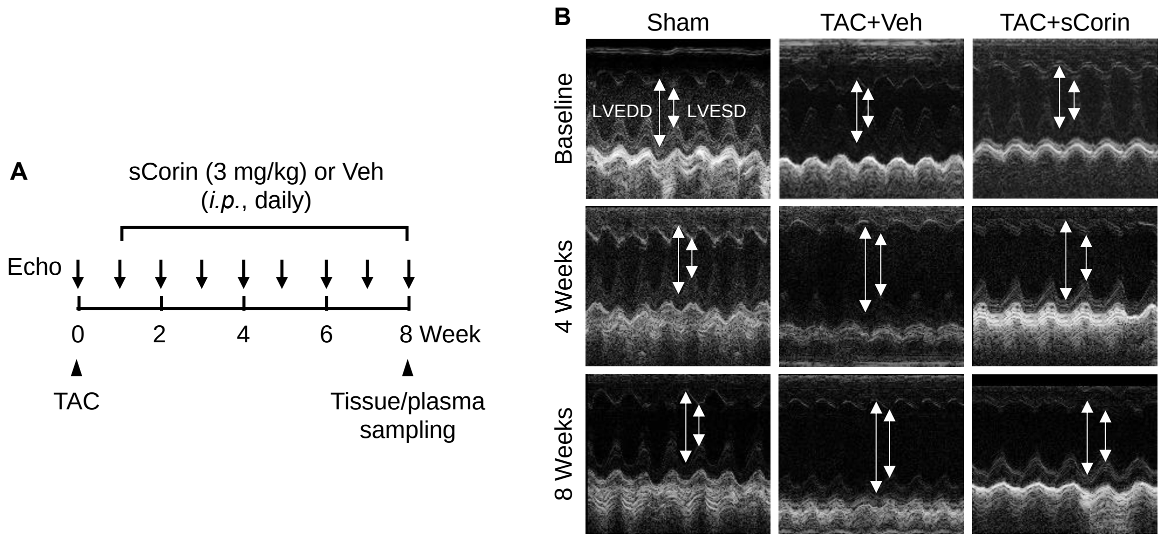

Supplementary Figure 3. Illustration of sCorin treatment in *Corin* KO mice subjected to TAC. (**A**) *Corin* KO mice (male, 10-12 weeks old) were subjected to the sham or TAC operation. One-week post-surgery, the TAC-operated *Corin* KO mice were treated with a vehicle (Veh) or sCorin (3 mg/kg) (*i.p.* daily). Echocardiography (Echo) was done weekly to assess cardiac function before (0 w) and up to eight weeks post-surgery. (**B**) Representative echocardiographic images from the sham- and TAC-operated *Corin* KO mice treated with vehicle or sCorin at 0- (baseline), 4-, and 8-weeks post-surgery.

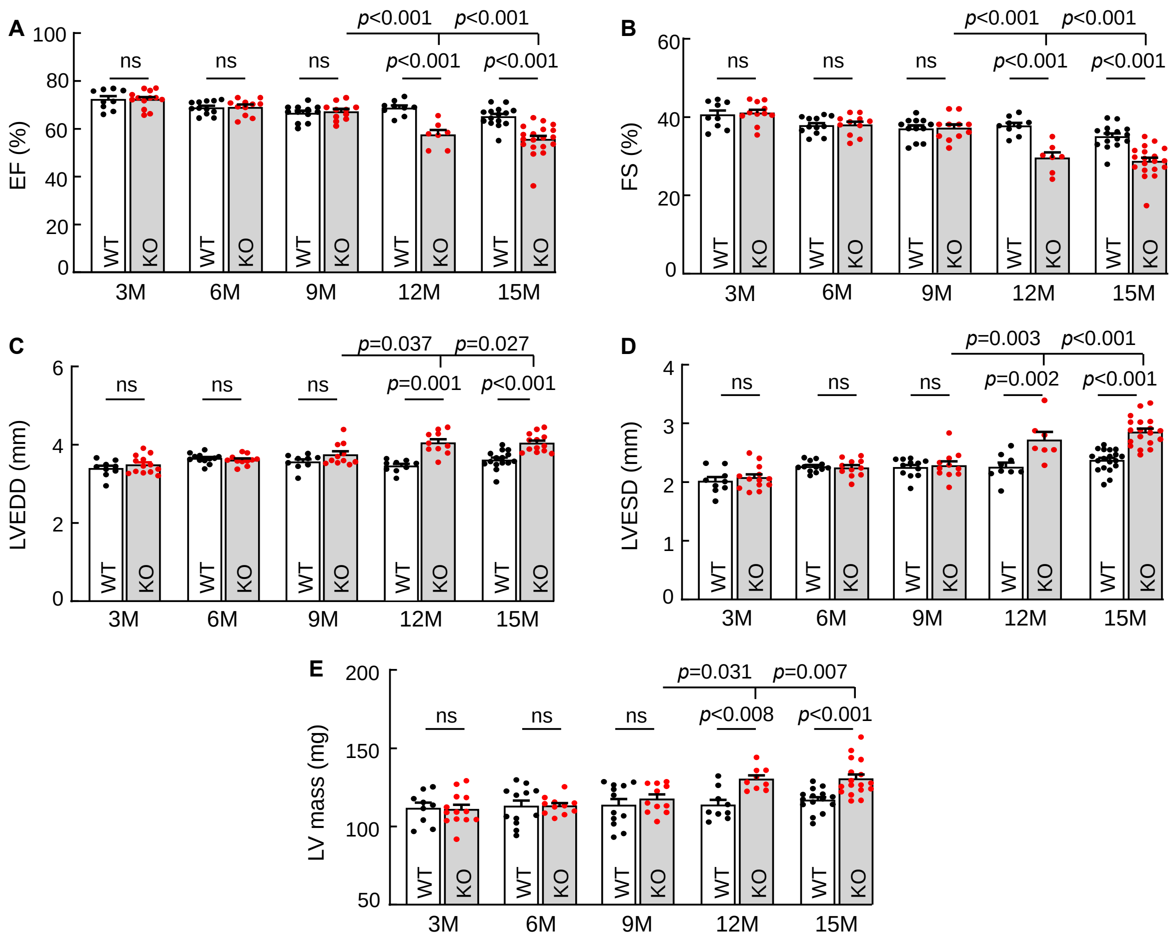

Supplementary Figure 4. Cardiac function in female WT and *Corin* KO mice at different ages. Echocardiography was conducted to examine ejection fraction (EF) (**A**), fractional shortening (FS) (**B**), left ventricular end diastolic dimension (LVEDD) (**C**), LV end systolic dimension (LVESD) (**D**), and LV mass (**E**) in female WT and *Corin* KO mice between 3-15 months (M) of age. Data are mean ± SEM; n = 7-19 per group. *P* values were analyzed by one-way ANOVA. ns: not significant.

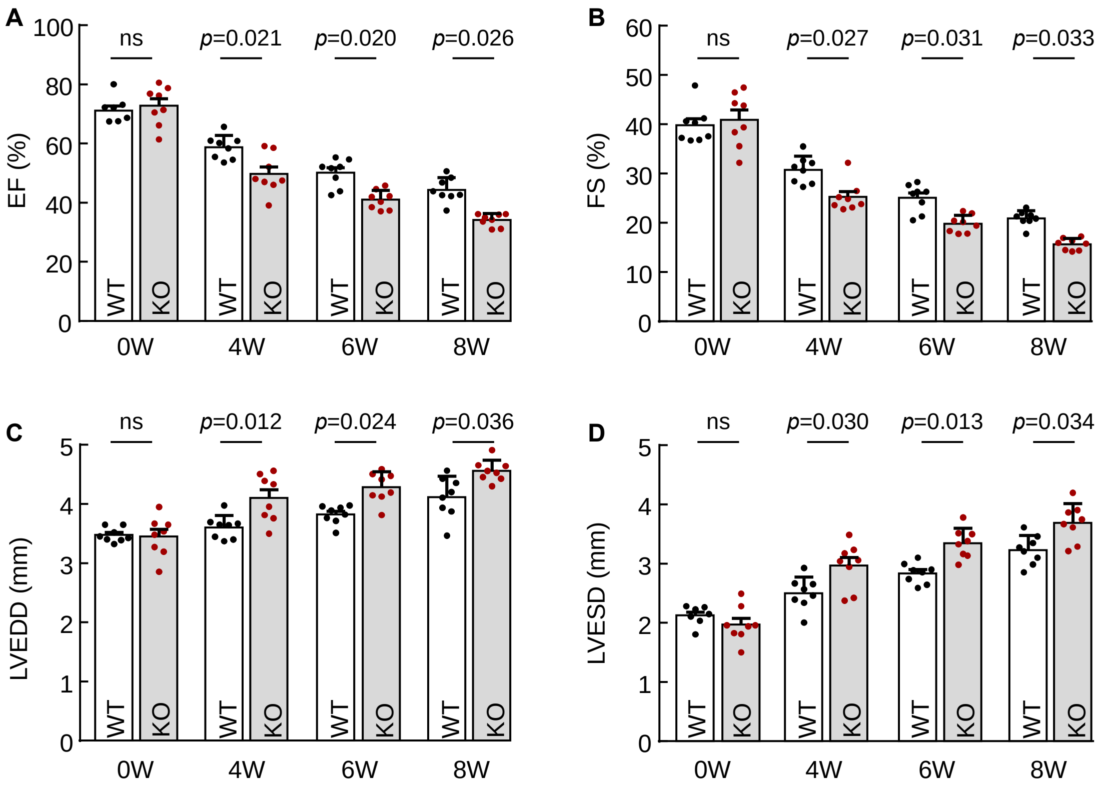

Supplementary Figure 5. Cardiac function in WT and *Corin* KO mice subjected to TAC. TAC was done in 10-12-week-old male WT and *Corin* KO mice. Cardiac function was assessed with echocardiography before (0 week) (W) and at 4, 6, and 8W post-surgery. The data of EF (**A**), FS (**B**), LVEDD (**C**), and LVESD (**D**) in the TAC-operated WT and *Corin* KO mice at different times were analyzed with two-way ANOVA. Data are mean ± SEM; n = 8 per group. ns: not significant.

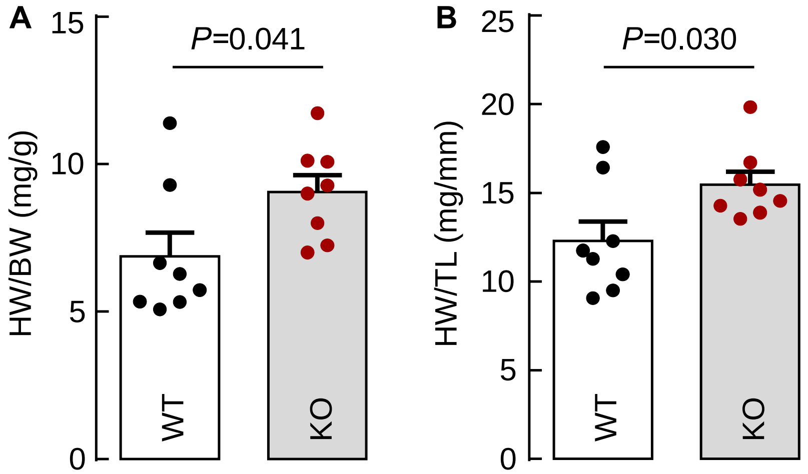

Supplementary Figure 6. Enhanced cardiac hypertrophy in *Corin* KO mice subjected to TAC. TAC was done in 10-12-week-old male WT and *Corin* KO mice. At 8 weeks post-TAC, hearts were isolated and weighed. Ratios of HW/BW (**A**) and HW/TL (**B**) in the WT and *Corin* KO mice were analyzed with Student's *t* test. Data are mean ± SEM; n = 8 per group.

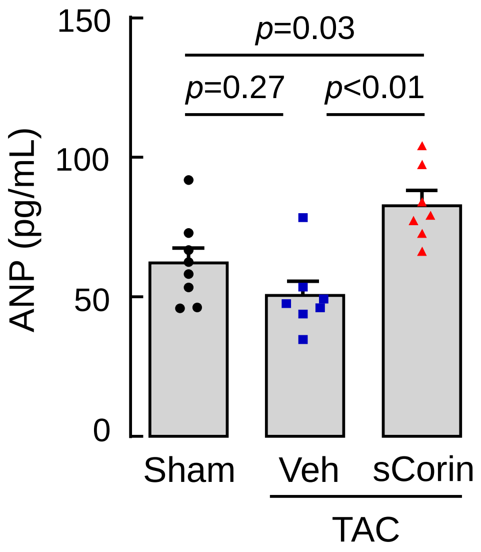

Supplementary Figure 7. Plasma ANP levels in sham- or TAC-operated *Corin* KO mice with vehicle or sCorin treatment. Plasma samples were isolated from the sham- or TAC-operated *Corin* KO mice with vehicle (TAC+Veh) or sCorin (TAC+sCorin) treatment at eight weeks post-surgery. Levels of ANP were measured by ELISA. Data are mean ± SEM. n = 7-8 per group. Data were analyzed by one-way ANOVA.

Supplementary Table 1. Sequences of the oligonucleotide primers used in this study.

Gene	Locus	Primer	Sequence	Size (bp)
Nppa	NM_008725.3	forward<br>reverse	CACAGATCTGATGGATTTCAAGA<br>CCTCATCTTCTACCGGCATC	68
Nppb	NM_001287348.2	forward<br>reverse	GTCAGTCGTTTGGGCTGTAAC<br>AGACCCAGGCAGAGTCAGAA	89
Myh7	NM_001361607.1	forward<br>reverse	GGCAAGCTCACATATACACAGC<br>ACAATCATGCCGTGCTGAC	111
Ctgf	NM_010217.2	forward<br>reverse	TGACCTGGAGGAAAACATTAAGA<br>AGCCCTGTATGTCTTCACACTG	112
Gapdh	NM_001289726.1	forward<br>reverse	TGTTCCTACCCCCAATGTGT<br>GGTCCTCAGTGTAGCCCAAG	137

Supplementary Table 2. Cardiac hypertrophy in *Corin* KO mice

	WT	*Corin* KO
Heart weight (mg)	161.87 ± 4.39	178.00 ± 2.43**
Body weight (g)	35.42 ± 1.09	34.09 ± 0.68
Tibia length (mm)	18.83 ± 0.14	18.81 ± 0.11

Hearts from 15-month-old male WT and *Corin* KO mice were weighed. Data are mean ± SEM. n = 15-16 per group. ***P* < 0.01 *vs*. WT, as analyzed by Student's *t* test.

Supplementary Table 3. Cardiac hypertrophy in TAC-operated *Corin* KO mice

	Sham	TAC
Heart weight (mg)	122.50 ± 4.53	225.00 ± 13.09**
Body weight (g)	25.66 ± 0.28	25.79 ± 0.27
Tibia length (mm)	16.40 ± 0.23	16.58 ± 0.69

Hearts from *Corin* KO mice at 8-weeks post sham or TAC operation were weighed. Data are mean ± SEM. n = 8 per group. **$P < 0.01$ *vs*. sham, as analyzed by Student's *t* test.

Supplementary Table 4. Cardiac hypertrophy in TAC-operated *Corin* KO mice treated with sCorin

	Sham	Vehicle	sCorin
Heart weight (mg)	136.00 ± 4.96	258.50 ± 14.84**	202.90 ± 10.35**$^{\#\#}$
Body weight (g)	26.34 ± 0.64	26.16 ± 0.48	25.83 ± 0.71
Tibia length (mm)	16.27 ± 0.14	15.84 ± 0.72	16.40 ± 0.56

Hearts from *Corin* KO mice at 8-weeks post sham or TAC with vehicle or sCorin treatment were weighed. Data are mean ± SEM. n = 13-15 per group. **$P < 0.01$ *vs*. sham; $^{\#\#}P < 0.01$ *vs*. vehicle, as analyzed by one-way ANOVA and Tukey's *post hoc* analysis.
